# Extra-pulmonary Cutaneous Sarcoidosis Presenting With Granulomatous Cranial Lesions and Cardiac Complications: A Case Report

**DOI:** 10.7759/cureus.53290

**Published:** 2024-01-31

**Authors:** Paul Q Vu, Prutha R Pathak, Siddharth Patel, Ashish K Basu, Mc Anto Antony, Amogh D Reddy, Jason Mathew

**Affiliations:** 1 Internal Medicine, Alabama College of Osteopathic Medicine, Dothan, USA; 2 Internal Medicine, North Alabama Medical Center, Florence, USA; 3 Internal Medicine, Decatur Morgan Hospital, Decatur, USA; 4 Cardiology, Heart Center, Decatur, USA; 5 Endocrinology, Diabetes, and Metabolism, Medical University of South Carolina, Anderson, USA

**Keywords:** high-grade atrioventricular block, complications of sarcoidosis, granulomatous skull bone lesions, cutaneous sarcoidosis, extra-pulmonary manifestations, granulomatous disorder, multisystemic sarcoidosis

## Abstract

Sarcoidosis is a non-caseating granulomatous disorder affecting multiple organs. Although the lungs are the most common site of presentation, extra-pulmonary manifestations involving the skin and heart can occur. Sarcoidosis affecting skull bone is uncommon and involvement of skin, heart, and skull bone all together, without pulmonary manifestations, is extremely rare. We report a 63-year-old Caucasian woman with a past history of cutaneous sarcoidosis and granulomatous skull bone lesions who presented with recurrent syncope. An ambulatory cardiac monitor detected intermittent high-grade atrioventricular block and cardiac MRI confirmed the diagnosis of cardiac sarcoidosis. This case represents an extremely unique journey of sarcoidosis and suggests potential consideration for cardiac sarcoidosis screening in patients with a history of extra-cardiac manifestations.

## Introduction

Sarcoidosis is a multisystem granulomatous disorder characterized by the formation of non-caseating granulomas [1]. Non-caseating granulomas are formed through a complex immune-mediated process driven by macrophages, T lymphocytes, monocytes, and regulatory B cells [2]. The incidence rate of sarcoidosis ranges from 7.6 to 8.8 per 100,000 person-years and has a bimodal age distribution [3-4]. Women are more likely to get sarcoidosis than men and it has a predilection towards African Americans compared to Caucasians [4]. The most common organs affected include lungs (>90%), eyes (>40%), skin (20-30%), and bone/joints (1-13%). Extra-pulmonary sarcoidosis in the absence of lung involvement is uncommon. Skull bone involvement is extremely rare. We present a 63-year-old Caucasian woman with cutaneous sarcoidosis who developed skull involvement and ultimately presented with cardiac sarcoidosis.

This article was previously presented as an e-poster at the Southern Medical Association Online Conference in August 2023.

## Case presentation

**Figure 1:**
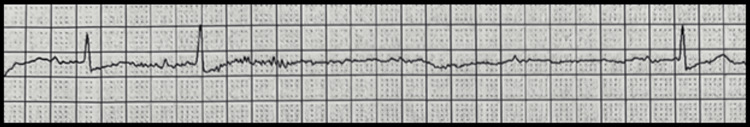
Event monitor (day 17) showing high-grade atrioventricular block

A 63-year-old Caucasian woman presented to the ER with intermittent episodes of transient loss of consciousness. The patient reported experiencing three similar episodes over the preceding four weeks. The first episode of loss of consciousness happened four weeks prior to the presentation when the patient was sitting in a recliner. It was sudden in onset without any warning signs: chest pain, palpitation, diaphoresis, jerky limb movements, incontinence, or tongue bite. The patient regained consciousness after about 45 seconds and had the recollection of all the events leading up to the episode. She also did not experience any confusion, headache, or change in vision after the event. She experienced another similar episode seven days later, after which an outpatient event monitor was arranged. The event monitor detected intermittent high-grade atrioventricular block around the time of her third episode at which point she was directed to go to the nearest ER (Figure 1). Noticeably, none of her episodes had any consistent association with body posture, diet consumed, or sleep pattern.

**Figure 2:**
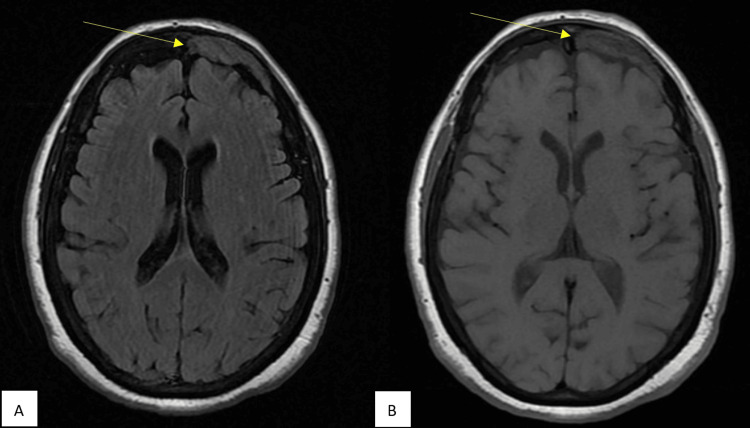
A 4.8 x 1.2 x 4.9 lesion involving left frontal skull bone and meninges of gadolinium enhancement on brain MRI (yellow arrow) (A) T2-weighted-Fluid-Attenuated Inversion Recovery (T2-FLAIR). (B) T1-weighted-conventional spin echo (T1-SE)

Her home medications included carvedilol twice a day and losartan for hypertension, pantoprazole for gastroesophageal reflux disorder, and simvastatin for hyperlipidemia. She had a known history of cutaneous sarcoidosis. MRI of the brain performed to investigate a transient ischemic attack two years ago detected lytic skull bone lesions, which were biopsied and indicated granulomatous inflammation (Figure 2). Apart from a hysterectomy for bleeding uterine fibroids, she did not have any other surgeries. She denied tobacco, alcohol, or other recreational substance use. She did not have a family history of sarcoidosis or other autoimmune disease. On initial physical examination in the ER, she had a temperature of 97.7 F, blood pressure of 170/80 mmHg, pulse of 66 per minute with regular rhythm, respirations of 20 per minute, and oxygen saturation of 95% on room air. Cardiovascular and respiratory examinations revealed normal S1 and S2 without murmur, rub or gallop and normal breath sounds without wheezing, rhonchi, or crackles, respectively.

**Figure 3:**
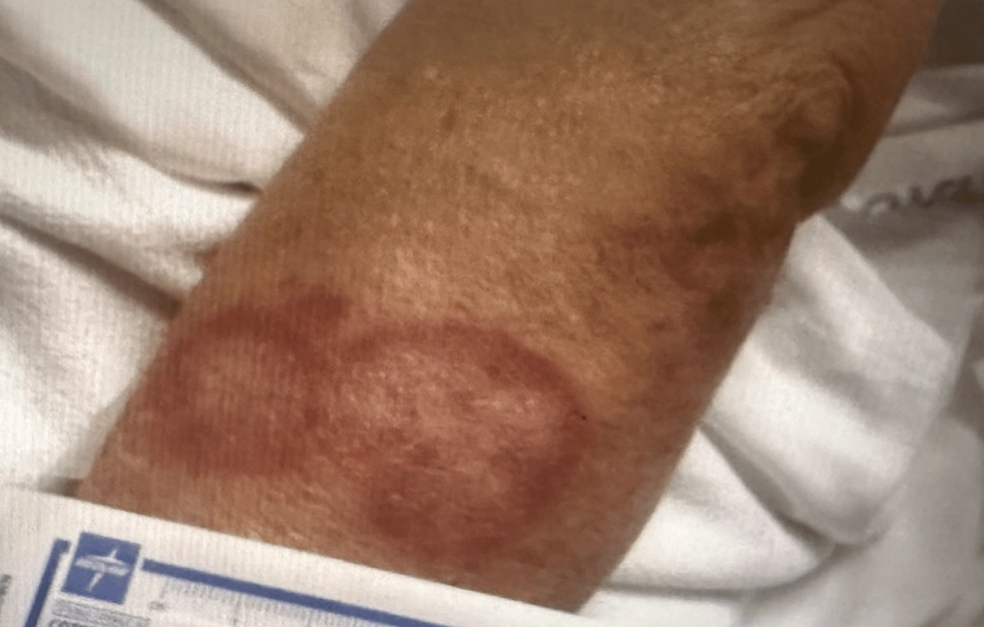
Discoid, erythematous, non-itchy patch distal to the right elbow

**Figure 4:**
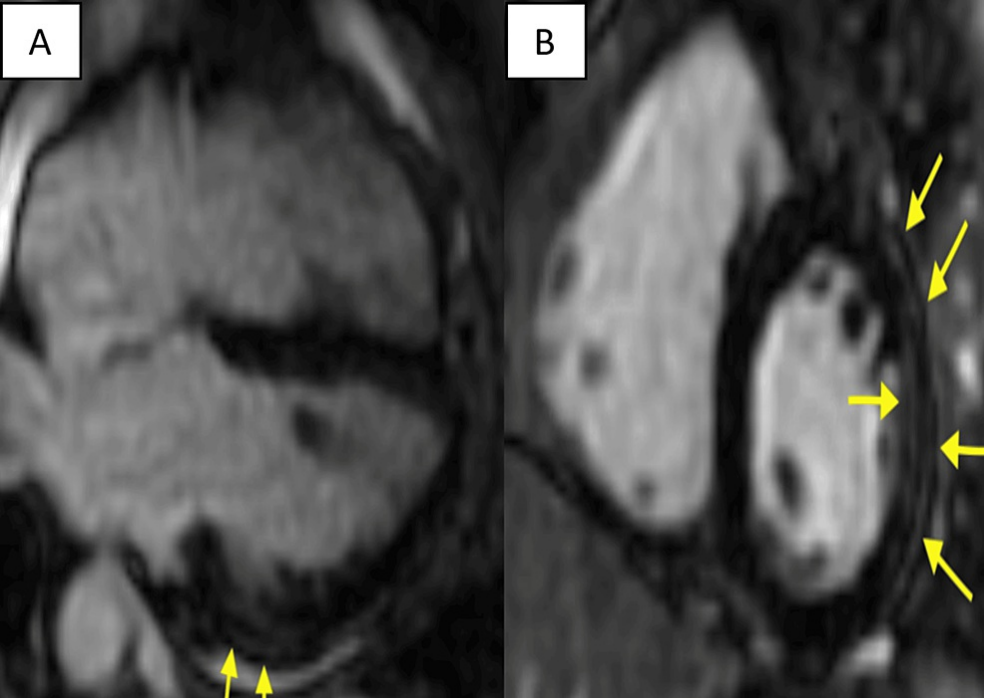
Multifocal areas of late gadolinium enhancement on cardiac MRI (yellow arrows) (A) Four-chamber view of post-contrast T1 images showing late gadolinium enhancement. (B) Short-axis view showing areas of late gadolinium enhancement

The patient did not have thyromegaly or cervical lymphadenopathy. A skin examination revealed discoid, erythematous, non-itchy patches affecting all extremities (Figure 3). Laboratory studies were obtained (Tables 1-2). Although, the event monitor showed an episode of intermittent high-grade atrioventricular block just a few hours prior to the patient’s presentation coinciding with her syncope, EKG in the ER showed a normal sinus rhythm. A chest x-ray did not show any abnormalities of the heart or lungs. The patient was admitted for overnight monitoring and carvedilol was discontinued. She did not experience a recurrence of heart block during her hospital stay. After discharge, a cardiac MRI confirmed the diagnosis of cardiac sarcoidosis (Figure 4). In addition to immunosuppressive therapy, the patient ultimately underwent an implantable cardiac defibrillator with pacemaker placement due to her high risk of developing dangerous arrhythmias, such as ventricular tachycardia and ventricular fibrillation.

**Table 1:** Blood investigations on admission

Test	Result	Reference range
WBC count	4.24 x 10^3 ^cells/mm3	4.8-10.8 x 10^3^ cells/mm3
RBC Count	4.66 x 10^6^ cells/mm^3^	4.7-6.1 x 10^6^ cells/mm^3^
Haemoglobin	14.2 g/dL	14.0-18.0 g/dL
Hematocrit	41.9%	42.0-52.0%
Mean corpuscular volume	89.9 FL	81-99 FL
Mean corpuscular haemoglobin	30.5 PG	27-31 PG
Mean corpuscular haemoglobin concentration	33.9 g/dL	33-37 g/dL
Red cell distribution width-standard deviation	12.7%	11.5-14.5%
Platelet count	168 x 10^3^ cells/mm^3^	130-400 x 10^3^ cells/mm^3^
Mean platelet volume	11.0 FL	7.4-10.4 FL
Sodium	142 mmol/L	136-145 mmol/L
Potassium	4.6 mmol/L	3.5-5.1 mmol/L
Chloride	107 mmol/L	88-107 mmol/L
Carbon dioxide	25 mmol/L	25-35 mmol/L
Anion gap	10 mmol/L	8-14 mmol/L
Blood urea nitrogen	16 mg/dL	8-22 mg/dL
Creatinine	0.9 mg/dL	0.7-1.2 mg/dL
Glucose	98 mg/dL	70-104 mg/dL
Calcium	8.8 mg/dL	8.8-10.2 mg/dL
Phosphorus	3.6 mg/dL	2.7-4.5 mg/dL
Magnesium	1.9 mg/dL	1.5-2.7 mg/dL
Total protein	7.1 g/dL	6.3-8.3 g/dL
Albumin	4.1 g/dL	3.5-5.0 g/dL
Total bilirubin	0.56 mg/dL	0.2-1.00 mg/dL
Aspartate aminotransferase	28 U/L	10-30 U/L
Alanine transaminase	17 U/L	10-36 U/L
Alkaline phosphatase	99 U/L	32-104 U/L
Prothrombin time	12.5 seconds	11.0-16.0 seconds
International normalized ratio	0.97	0.9-1.1
Partial thromboplastin time	26.4 seconds	22.3-41.8 seconds
D-dimer	1.71 mg/mL FEU	0.0-0.52 mg/mL FEU

**Table 2:** Angiotensin-converting enzyme and immunological studies upon admission

Test	Result	Reference range
Angiotensin-converting enzyme	49 U/L	16-85 U/L
Anti-nuclear antibodies	Negative	Negative
IgA, serum	175 mg/dL	70-400 mg/dL
IgG, serum	1198 mg/dL	700-1600 mg/dL
IgM, serum	76 mg/dL	40-230 mg/dL

## Discussion

**Figure 5:**
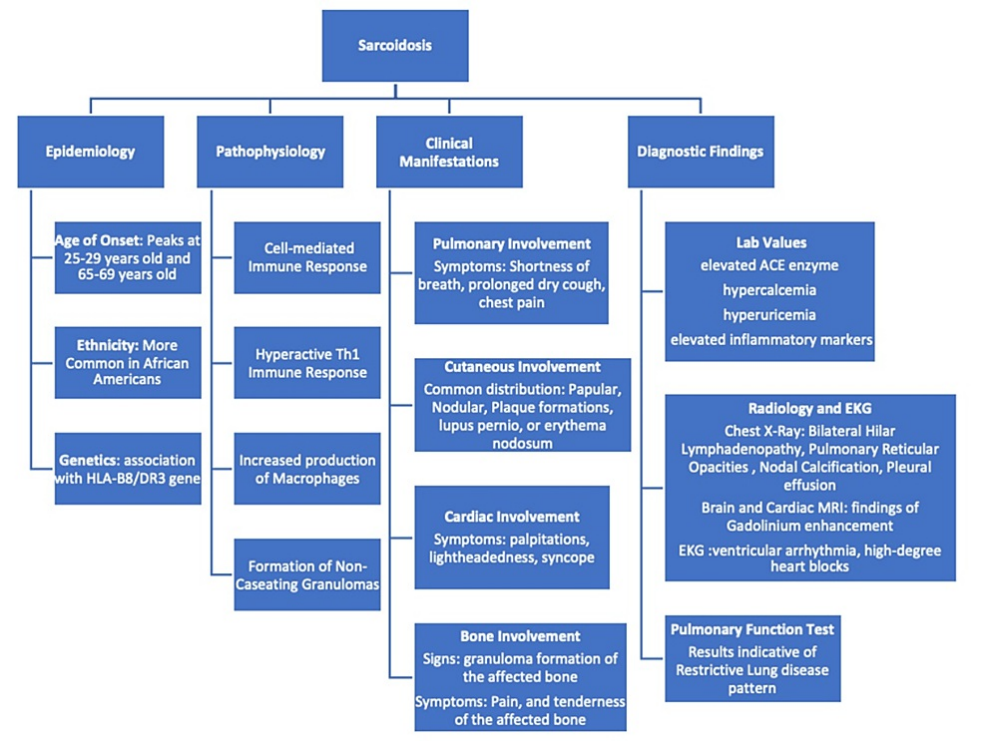
Overview diagrammatic representation of sarcoidosis

Sarcoidosis is a rare multi-system disorder characterized by the development of non-caseating granulomas (Figure 5). The formation of non-caseating granulomas is a Type 1 T helper (Th1) immune-mediated response involving IL-2, interferon-gamma (IFN-γ), and tumour necrosis factor-alpha (TNF-α) [5]. From 2010-2013, the overall average incidence of sarcoidosis in the United States was 8.275 per 100,000 [4]. There are two peaks of incidence for women aged 25-29 years (10.5%) and 65-69 years (11.0%) [6]. There is a higher prevalence of sarcoidosis in African American individuals (141.6 per 100,000 people) compared to White individuals (49.8 per 100,000) with the highest prevalence being in African-American women (178 per 100,000) [1,4]. Interestingly, there is a possible genetic link to sarcoidosis in patients carrying the HLA-B8/DR3 haplotype [7]. Pulmonary sarcoidosis is the most common manifestation (95%) which presents with cough, dyspnea, and chest pain. Pulmonary function tests (PFTs) usually demonstrate a restrictive lung disease pattern, and chest X-ray shows reticular opacities with bilateral hilar adenopathy [8]. Also, with certain clinical findings such as Lofgren’s syndrome (erythema nodosum, hilar adenopathy, migratory polyarthralgia, and fever) and Heerfordt syndrome/uveoparotid fever (anterior uveitis, parotid gland enlargement, facial nerve palsy, and fever), a clinical diagnosis of sarcoidosis can be made [9-10]. However, the diagnosis of sarcoidosis is more difficult if pulmonary findings are not present initially as in our case, since extra-pulmonary presentations of sarcoidosis are uncommon (31.4%) [11].

Our patient, a 63-year-old Caucasian woman, did not have a cough or dyspnea and had a normal chest x-ray. She presented with cutaneous sarcoidosis (15.9%) that eventually affected skull bone (0.5%), and heart (2.3%) [4]. Furthermore, cutaneous sarcoidosis, which is the most common extra-pulmonary presentation, typically presents as papular, nodular, plaque-like lesions (e.g. lupus pernio, or erythema nodosum) [12], whereas our patient had discoid, erythematous, non-itchy patches affecting all extremities. Additionally, subgroups of sarcoidosis have been described before (e.g. ocular-cardiac-cutaneous-central nervous system disease involvement, musculoskeletal-cutaneous involvement, pulmonary and intrathoracic lymph node involvement) but a new subgroup may be identified as our patient had cutaneous-musculoskeletal-cardiac involvement [13].

In our case, she had cutaneous sarcoidosis and eventually developed non-caseating granulomas involving the skull bone and heart (incidence of three-organ-involvement, 18.7%) [14]. Therefore, patients who present with cutaneous sarcoidosis may benefit from early screening of other organ systems (e.g., continuous ambulatory EKG or echocardiography to monitor cardiac involvement) [15]. Due to the possibility of severe consequences of cardiac sarcoidosis (e.g., ventricular arrhythmia, high-grade heart blocks, and progressive heart failure), early detection and diagnosis are of high priority to prevent mortality [16].

## Conclusions

Sarcoidosis is a complex granulomatous disorder that affects multiple organ systems. Pulmonary sarcoidosis is the most common presentation and extra-pulmonary sarcoidosis without lung involvement can occur but is extremely rare. Cutaneous sarcoidosis should raise a high degree of suspicion and possibly initiate early screening for other organ system involvement regardless of symptoms. The failure of timely diagnosis and therapy may result in progressive organ system involvement with potential life-threatening consequences.
